# Terahertz detectors arrays based on orderly aligned InN nanowires

**DOI:** 10.1038/srep13199

**Published:** 2015-08-20

**Authors:** Xuechen Chen, Huiqiang Liu, Qiuguo Li, Hao Chen, Rufang Peng, Sheng Chu, Binbin Cheng

**Affiliations:** 1 State Key Laboratory of Optoelectronic Materials and Technology, Sun Yat-Sen University, Guangdong Guangzhou 510275, China; 2 School of Mobile Information Engineering, Sun Yat-Sen University, Guangdong Guangzhou 510275, China; 3 State Key Laboratory Cultivation Base for Nonmetal Composites and Functional Materials, Southwest University of Science and Technology, Sichuan Mianyang 621010, China; 4 Institute of Electrical Engineering, Chinese Academy of Engineering Physics, Mianyang 621423, China

## Abstract

Nanostructured terahertz detectors employing a single semiconducting nanowire or graphene sheet have recently generated considerable interest as an alternative to existing THz technologies, for their merit on the ease of fabrication and above-room-temperature operation. However, the lack of alignment in nanostructure device hindered their potential toward practical applications. The present work reports ordered terahertz detectors arrays based on neatly aligned InN nanowires. The InN nanostructures (nanowires and nano-necklaces) were achieved by chemical vapor deposition growth, and then InN nanowires were successfully transferred and aligned into micrometer-sized groups by a “transfer-printing” method. Field effect transistors on aligned nanowires were fabricated and tested for terahertz detection purpose. The detector showed good photoresponse as well as low noise level. Besides, dense arrays of such detectors were also fabricated, which rendered a peak responsivity of 1.1 V/W from 7 detectors connected in series.

The terahertz (THz) radiation region is defined by the frequency range of 0.1 to 10 THz. This range contains abundant physical and chemical information of most substances, making great benefits to vast applications such as interstellar observation, homeland security, as well as medical diagnosis^1^,^2^. Under this context, much effort has been devoted to achieve high-efficiency, low-cost, and room temperature THz sources and detectors. On the source side, recently miniaturized THz quantum cascade lasers (QCL)^3^ and photoconductive THz emitters^4^,^5^ have shown good progress in both output power and operation temperature.

On the detector side, there already exist many THz detection solutions, such as bolometers^6^, Schottky diode detectors^7^, and field effect transistors (FETs), etc. Among them, plasmon wave detection based on FETs^8^,^9^ possesses several advantages in terms of room temperature operation, wide detection range, and compactness for light-weight on-chip array technologies. In this case, recently a novel approach for simple structured FET detectors by integrating semiconductor nanowires (or graphene) as active elements^10^ has been developed and showed good sign of promise. In those devices, a non-resonant broad band detection mechanism often arises because plasma oscillations are normally overdamped in the nanowire channel (*ω*_0_*τ* ≪ 1, where *ω*_0_ is the fundamental plasma frequency and *τ* is the momentum relaxation time)^11^, which can lead to a measurable signal between source and drain electrodes.

Several nanomaterials have been fabricated into THz FET detectors, including InAs^11^,^12^, InSb^13^, graphene^14^, and ZnO^15^. InN thin films and nanowires, on the other hand, are considered as promising candidate for efficient THz emitters (photo-Dember type) due to their high peak/saturation drift velocity, and low intervalley scattering rate^16^. In fact, InN has theoretical electron mobility up to 14000 cm^2^/VS^17^, which makes it in principle suitable for THz applications. Hence the development of THz detectors based on InN nanowires is of strong interest. Here in this contribution, an systematic investigation was made on the material synthesis, nanowire alignment, device fabrication, device arrays integration and THz characterizations for InN nanomaterial. On the growth section, it is found that during InN nanowire growth, another material form nano-necklace was formed simultaneously. Then the crystal properties and growth mechanisms were studied in detail. For device demonstration, especially instead of conventional single nanowire device geometry, THz detectors were fabricated from several aligned nanowires. THz detectors array was further realized by integration individual nanowires-device in series. The advantages of this approach lies in: 1. only nanowires with controlled density, position, as well as orientation will likely find ultimate applications. 2. THz wave has typical focus spot larger than 100 μm. If more than one subwavelength sized devices are connected, stronger photoresponse can be generated. Hence in the fabrication process we adopted a “transfer printing” approach developed by others^18^,^19^, which enabled alignment of groups consist 3 to 6 InN nanowires into designed areas for subsequent device fabrication. The result of these devices unequivocally demonstrates fairly good FET properties of the multi-nanowires sample, which successfully produced sum responsivity of 1.1 V/W from integrated device arrays. This result is important for many THz applications.

## Results

**Figure 1:**
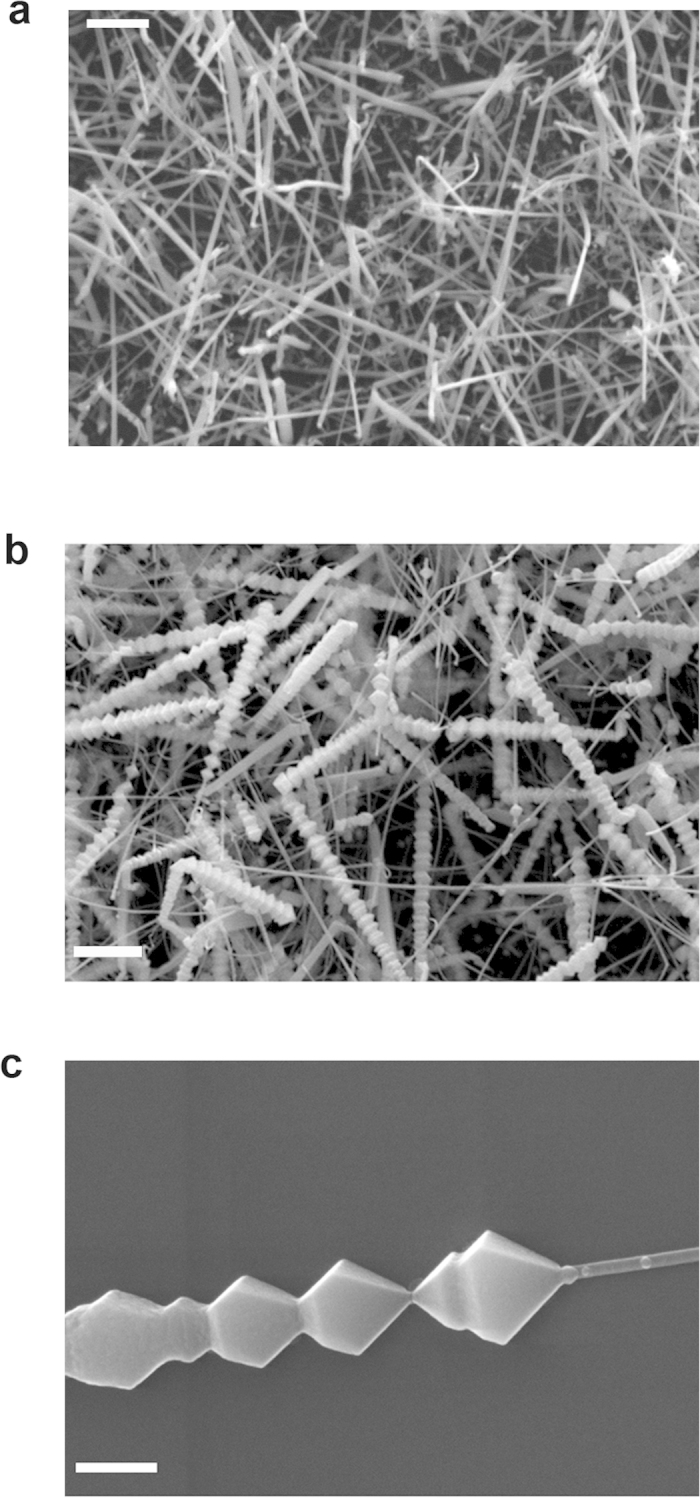
FESEM images of the nanowire and nano-necklace structures. (**a**) scale bar: 2 μm. (**b**) scale bar: 1 μm. (**c**) Magnified SEM images of a typical InN nano-necklace architectures, scale bar: 300 nm.

The InN nanostructures were prepared by chemical vapor deposition (CVD) method. Although the growth was intentionally designed for only nanowires, however, distinctly, after growth there were two kinds of InN morphologies seen on the as-grown substrate: nanowires and “nano-necklace”. It is then found that the places close to the source grow nanowires whereas further away positions were covered mostly by nano-necklace. Fig. 1a,b are the scanning electron microscope (SEM) images of InN nanowires and nano-necklaces on close and far parts (to the source) of the substrate, respectively. The co-existence of different morphologies suggests that the growth of various InN nanostructures in CVD has narrow growth windows. Thus, slight variation of growth parameter (such as III/V ratio) can yield drastic different growth modes. In addition, the heads or parts of these nanomaterials do not contain gold nanoparticles (method section) in SEM images, indicating the vapor-solid growth mode^20^. The role of gold nanoparticles prior to the growth is to increase the surface roughness to facilitate vapor nucleation. Magnified SEM image of a typical InN nano-necklace architecture is shown in Fig. 1c, which exhibits clearly that the typical nano-necklace consists of a number of connected and uniform beads. The major and minor waists range from 200 to 400 and 200 to 1000 nm, respectively.

**Figure 2:**
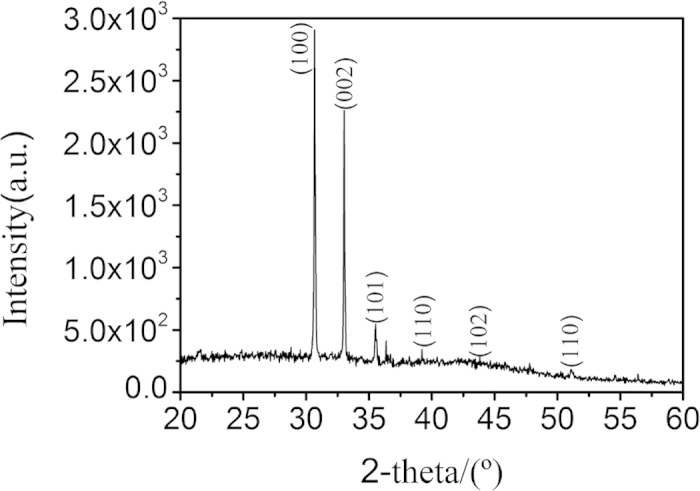
XRD pattern taken on the as-grown InN nanostructures on Si substrate.

**Figure 3:**
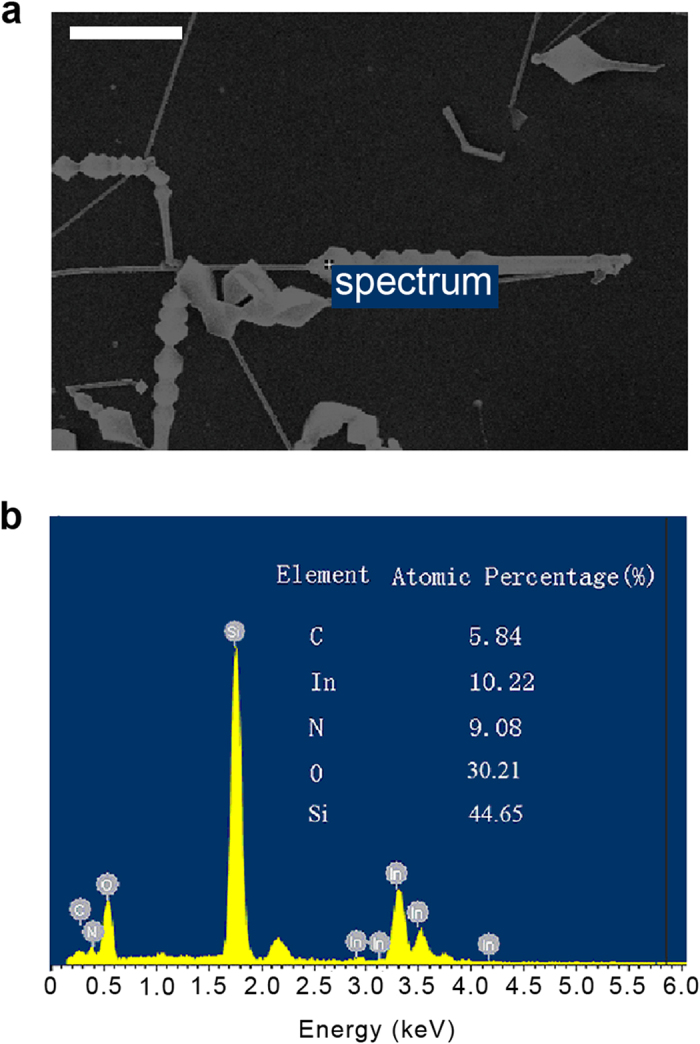
(**a**) SEM image of as-synthesized InN nanostructures. scale bar: 1 μm. (**b**) The corresponding EDS spectrum of the InN nanostructures taken at spot spectrum 9 which are marked in (**a**). Inset: the corresponding composition of the nanostructures determined by EDS.

The crystal structure of the as-obtained product was characterized by X-ray diffraction (XRD) (Fig. 2). All of the strong reflection peaks can be indexed to wurtzite-type InN (w-InN). According to the Bragg equation (2*d*sin*θ *= *λ*) and interplanar spacing formula 
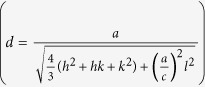
, where *d*, *θ*, *λ*, {*hkl*} are the interplanar spacing, diffraction angle, wavelength, indices of crystal face, respectively. The lattice constants can be calculated as: a = 0.35 nm and c = 0.57 nm (JCPDS card NO. 02-1450), which match well with the (100) and (002) planes of w-InN InN, respectively^21^. XRD peaks related to other phases (such as In and In_2_O_3_) were not found. Energy dispersive spectroscopy (EDS) measurements were performed to determine the chemical composition of the samples. Fig. 3a illustrates a typical SEM image of sample part that found co-existence of InN nano-necklaces and nanowires. The EDS spectrum of marked position in Fig. 3a is shown in Fig. 3b. Distinct In and N peaks were found to confirm the InN elemental composition, while the silicon peak originates from the substrate. Oxygen peak is attributed to SiO_2_ and Indium oxide related substances. The atomic ratio of In: N is close to 1 : 1, suggesting good stoichiometry of the material.

**Figure 4:**
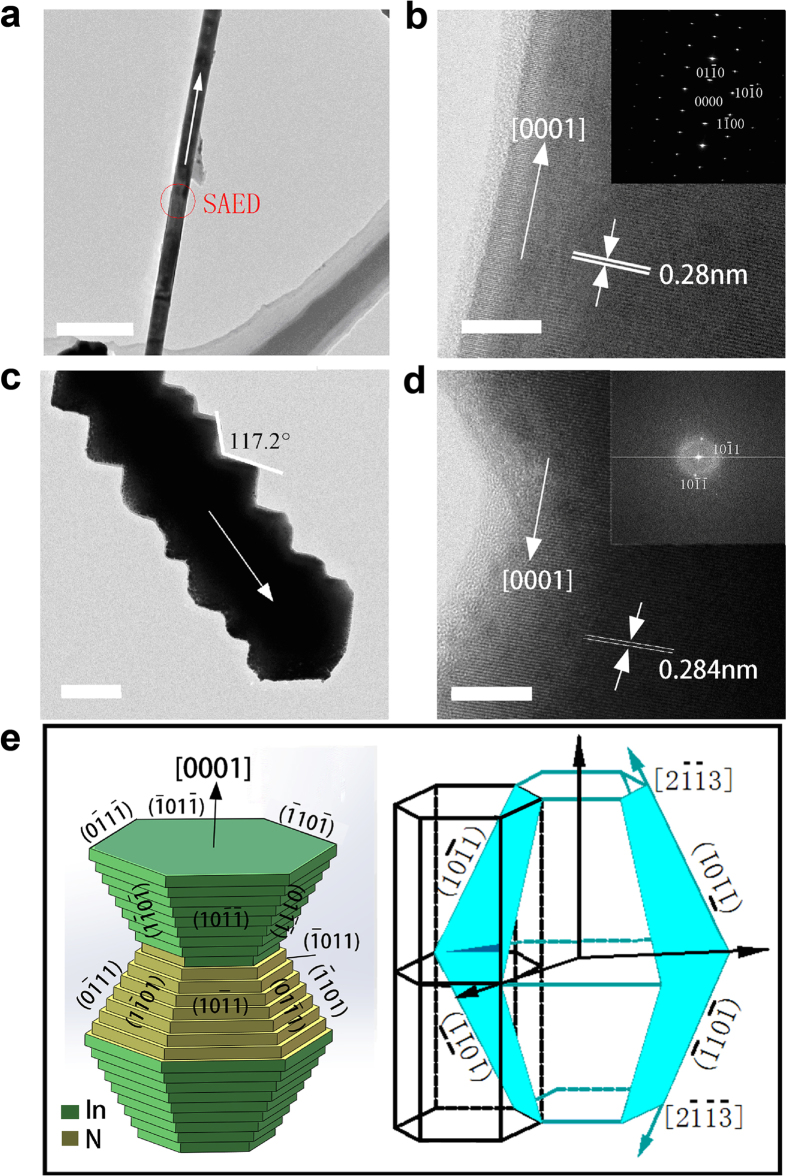
TEM and HRTEM images of the InN nanowire and InN nano-necklace. (**a**) TEM image of the individual nanowire, scale bar: 500 nm, (**b**) The corresponding HRTEM image and SAED pattern of the nanowire, scale bar: 10 nm. (**c**) TEM image of the individual nano-necklace, scale bar: 100 nm, (**d**) The corresponding HRTEM image and FFT pattern of the nano-necklace, scale bar: 10 nm. (**e**) Space-filling model of ideal [0001] nano-necklace and schematic of a crystal unit of the w-InN with 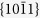 and {0001} planes enclosed.

The chemical composition and microstructure of as-synthesized InN nanostructures were further studied by transmission electron microscope (TEM). The TEM images of single InN nanowire and nano-necklace sample are shown in Fig. 4a,c, respectively. High resolution (HR)-TEM of Fig. 4b is the magnified part of the single nanowire to examine detailed crystal structure. The d-spacing of the lattice fringes measured is 0.28 nm, matching well with the (0001) plane interspacing of wurtzite InN structure. The inset of Fig. 4b shows the corresponding selected area electron diffraction (SAED) pattern taken from the corresponding nanowire, further confirming that the sample is single-crystalline and grows preferentially along the [0001] crystallographic direction. For the InN nano-necklace, Fig. 4c indicates that the nano-necklace consists of multiple single crystalline connected beads, while no grain boundaries between them could be noticed. HRTEM of Fig. 4d (also with fast fourier transfor (FFT) in the inset of Fig. 4d) shows fringe with d-spaces of 0.31 nm. After the TEM studies, the growth pattern of the nano-necklaces can be deduced by the assistance from a w-InN unit cell (Fig. 4e): the growth direction of the nano-necklace is determined to be also [0001]; the equilateral trapezoid facets of the truncated hexagonal beads belong to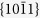; the edges between two facets are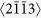. By using the standard lattice parameters of w-InN, the angle between two corresponding facets on the opposite sides of the major waist of the beads (e.g.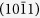 and 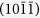 is calculated to be 124° and that between two corresponding edges (e.g. 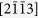 and 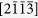) to be 116°. These are in good agreement with the observed values ranging from 116° to 124° in Fig. 4c. The schematic illustration of the nano-necklace is presented in Fig 4e. The fractions of 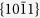 and 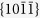 planes are not equal, which may owes to the different polarities of them: the former is In-polar while the later is N-terminated. In other words, the as-obtained nano-necklace (see Figs 1 and 4c) are not exactly the same as simulated Fig. 4e; instead, the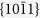 planes are more favored than the 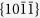 ones, which is due to the different formation energies thus different diffusion abilities of the adatoms on these two types of side-surfaces^22^. In addition, similar multi-beads morphologies are also observed in one dimensional hexagonal wurtzite stacked-cone and zigzag AlN^23^, GaN^24^ and ZnO^25^ nanostructures.

**Figure 5:**
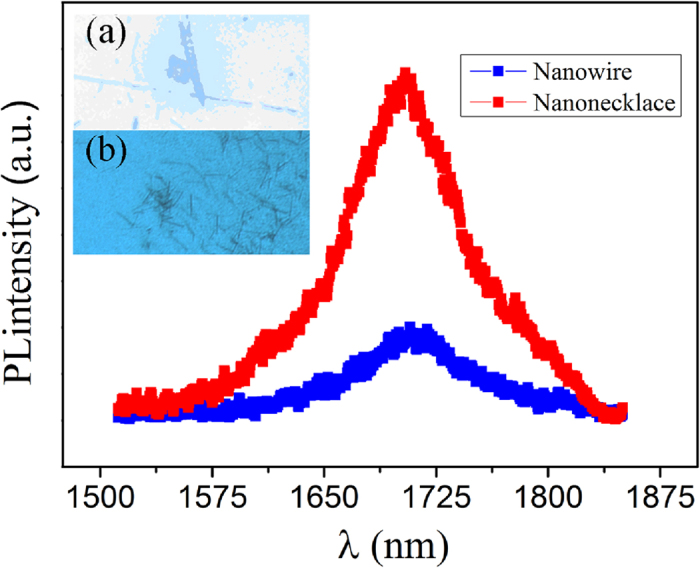
PL spectra InN nanostructures at room temperature. The inset shows microscope images of the InN nano-necklaces and nanowires.

The photoluminescence (PL) of the two kinds of structures was carried out (Fig. 5) to investigate the optical properties of single InN nanowire and nano-necklace. The strong emission peaks around 1700 nm can be seen clearly, which corresponds to the near band edge (NBE) emission of InN. This IR-emission peak position is consistent with the NBE emission observed from reported InN nanorods^26^ and epitaxial films^27^. Notefully, the emission intensity from the nano-necklace is stronger, and is tentatively attributed to the larger material size as well as muti-scattering inside one nano-necklace for three dimensional cavity-like light resonant^28^.

**Figure 6:**
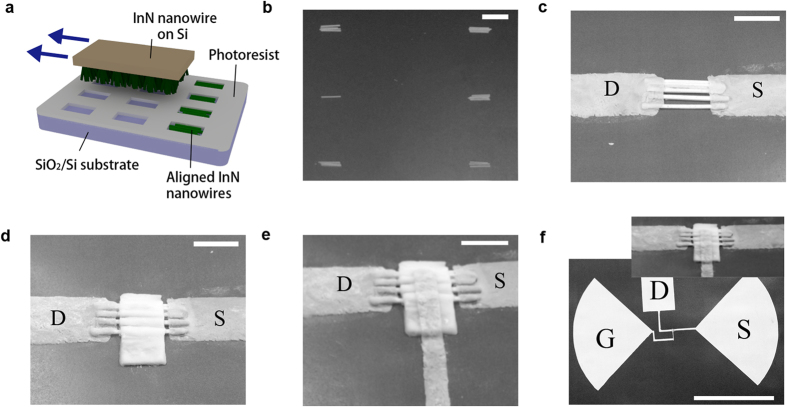
(**a**) Schematic drawing of the “transfer printing” process. (**b**) SEM image of InN nanowires in array groups derived from “transfer printing” process. Scale bar: 5 μm. (**c**) SEM image of the aligned nanowires with source and drain electrodes deposited. Scale bar: 2 μm. (**d**) SEM image of the nanowire device with gate 300 nm Al_2_O_3_ deposited. (**e**) SEM image of the nanowire FET based on 5 aligned nanowires. Scale bar: 2 μm. (**f**) SEM image of the whole device. Scale bar: 100 μm.

Although single InN nanowire or nano-necklace can be readily used for THz detectors such like single nanowire FET detectors, randomly distributed nanostructures are less favorable for ultimate practical applications. For simplicity and wider impact purpose, InN nanowires rather than nano-necklaces were selected to perform alignment and device fabrications. They were transferred from growth Si substrates to a patterned device substrates by a mechanical “transfer printing” process (Fig. 6a). The alignment direction is determined by the mechanic shear force due to relative motion of the two substrates. SEM image of Fig. 6b indicates that 3 ~ 6 nanowires can be successfully transferred into trench arrays after the transfer-printing process. In the following FET device fabrication, one group containing 5 nanowires was selected for demonstration. Source, drain (10 nm Ti/20 nm Au) were defined and deposited by photolithography and e-beam evaporation (Fig. 6c) to two heads and tails of the nanowires. The gate area was defined by photolithography and then 300 nm thick Al_2_O_3_ gate insulator was deposited by e-beam evaporation (Fig. 6d). The gate electrode (50 nm Pt) is finished by the assistance of focused ion beam (FIB) technique (Fig. 6e). Bow-shaped antenna with radius of 100 μm was designed to partially resonant with incidence THz wave, while the left and right lobes of the antenna were connected to source and gate electrodes, respectively. The final device structure is also seen in Fig. 6f. The reason of the typical un-symmetric source-drain electrode is for rectifying enhancement to maximize the photovoltage readout.

**Figure 7:**
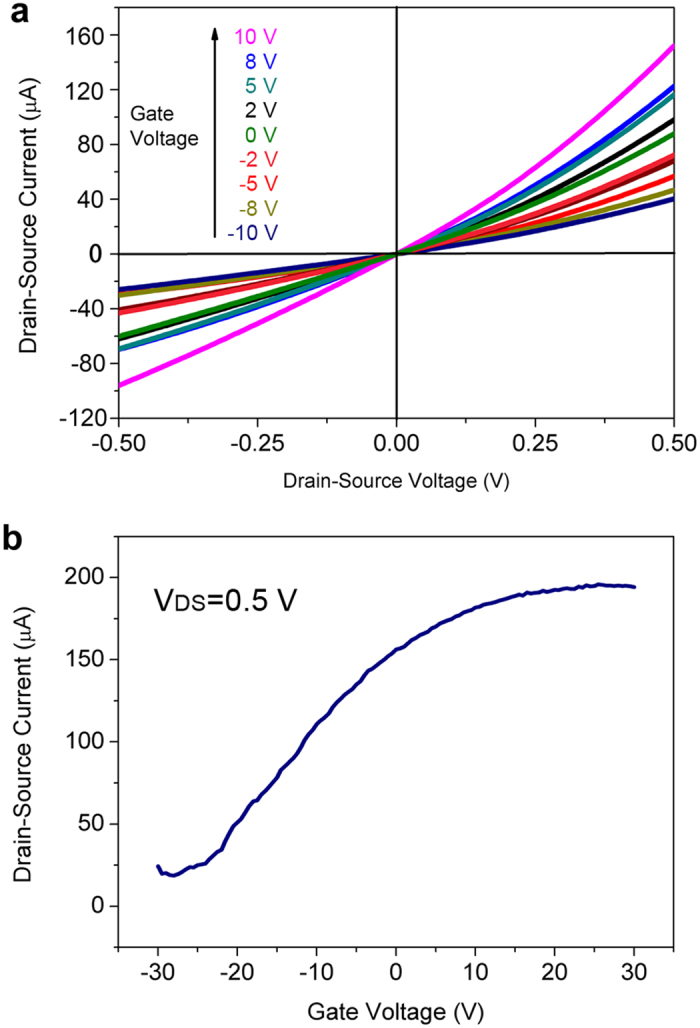
(**a**) I_DS_ vs V_DS_ curve of the FET device under different V_G_. (**b**) I_DS_ vs V_DS_ curve when V_DS_ kept at 0.5 V.

With the above device geometry, FET characterizations on a single array which consists of 5 InN nanowires were performed at room temperature. The source-drain current (I_DS_) versus source-drain voltage (V_DS_) curves under different gate voltages (V_G_) −10 V to 10 V is shown in Fig. 7a. I_DS_ increases with the increase of V_G_ evidently, suggesting typical n-type channel characteristics^29^. Fig. 7b shows the I_DS_-V_G_ transfer curve at V_DS_ = 0.5 V. It is observed that the I_DS_ increases with the increase of V_G_ from −30 V to 30 V, which is in accordance with Fig. 7a. It is notes that with high V_G_, I_DS_ increase becomes nonlinear, which is possibly due to high current (~ 30 μA in one nanowire) annealing effect. Also the corresponding I_DS_ at given V_G_ is not well matched between Fig 7a,b, which is attributed to the hysteresis behavior of the I_DS_-V_G_ curve. Unlike ZnO nanowire FETs^29^ or carbon nanotubes^30^, the device does not exhibit very clear and high on/off ratio. The reason is due to residue carrier density that not swept by V_G_. The threshold voltage (V_th_) is found to be about −25 V ~ −30 V. In a nanowire FET, the carrier concentration *n* can be estimated by using the following equation^31^:
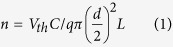
 where L, d, V_th_, and q are the channel length (~ 1 μm), the nanowire diameter (averagely 200 nm), the threshold voltage of the nanowire FET (equals to −25 ~ −30 V), and the elementary charge constant q = −1.6 × 10^−19^ C, respectively. C is the capacitance of the FET device, which is modeled by electrostatic module of Comsol Multiphysics (COMSOL Inc.) to be 4.07 × 10^17^ F. It is assumed that InN nanowires are with similar electrical properties and it can be assumed that the V_th_ are the same for the analysis. From the equation, the electron concentration is calculated to be 3.64 ~ 4.36 × 10^17^ cm^−3^. The mobility can be calculated through the following equation^31^:
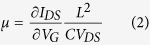
 where L = 1 μm is the channel length (gate length); V_DS_ = 0.5 V; transconductance 
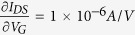
 from the linear region of the I_DS_-V_G_ curve. After all, the mobility of 99 cm^2^/VS could be calculated by equation (2), which is on the same order with reported InAs nanowire THz detectors (~ 500 cm^2^/VS)^32^.

## Discussion

**Figure 8:**
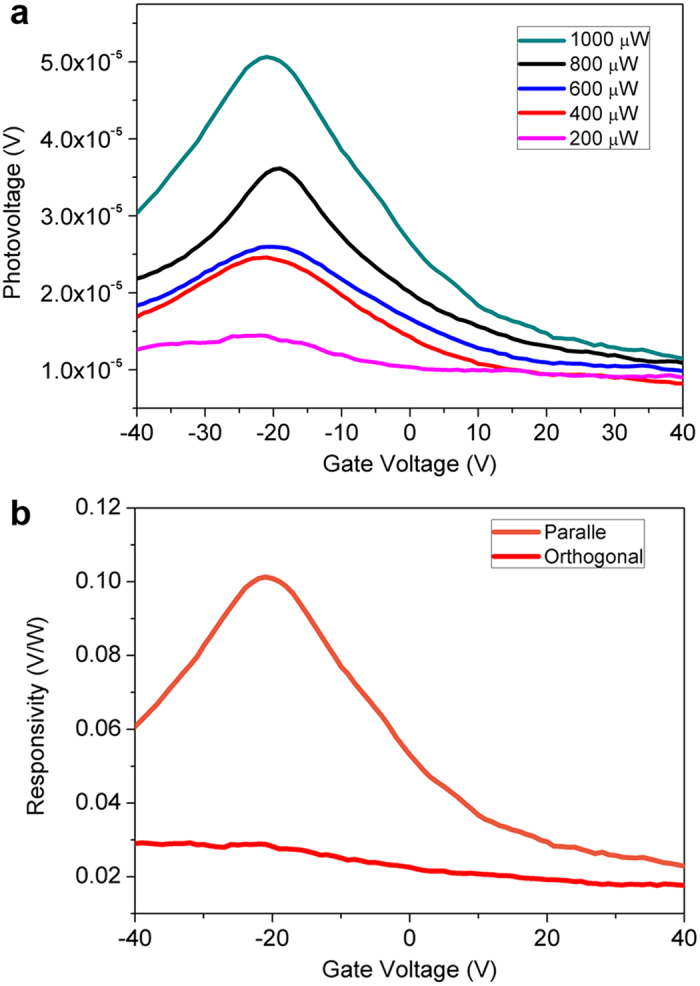
(**a**) THz photovoltage as a function of gate voltage under different incidence power. (**b**) Responsivity curves when incidence polarization is paralle (orange curve) or orthogonal (red curve) respect to the antenna.

The THz induced photovoltage vs V_G_ is depicted in in Fig. 8a. It is observed that there are obvious peaks and the maximums of the photovoltage happen around −20 ~ −22 V, which are in proximity with V_th_ in I_DS_-V_G_ curves. Analysis suggests that this behavior is in accordance with the detection mechanism of a plasmon wave FET detector^33^, in which the source-drain photovoltage can be expressed as: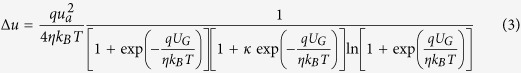 Where *q* is the electron charge, *u*_*a*_ is the small amplitude of the ac wave, *η* is the ideality factor, *k*_*B*_ is the Boltzmann constant, *T* is the temperature and U_G_ denotes the gate-to-source voltage minus *V*_*th*_ (U_G_ = V_G_−V_th_), and 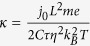 (here J_0_ is the gate leakage current density, *L* is the channel length, *m* is the electron mass, *C* is the channel capacitance per unit area, *τ* is the momentum relaxation time). Under this circumstance, the maximum of the photovoltage: 
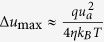
 can be derived at the condition when *U*_*G*_ = −(*ηk*_*B*_*T*/2)ln(1/*κ*). For different J_0_, U_G_ can be on the range of 0 ~ −5 V^33^. However precise value of maximum point U_G_ could not be given due to other un-available parameters. Nevertheless, the maximum U_G_ happens around the point *V*_*G*_ ≈ *V*_*th*_ with several volts offset, which holds good accordance in the current device data in Fig. 8a. Further, in order to get responsivity values, the following geometric relationship should be taken into account:
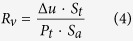
 where R_V_ is the responsivity, P_t_ is the incidence power impinging on the beam spot, S_t_ is the radiation beam spot area and S_a_ is the active area. S_t_ = πd^2^/4 = 1.77 × 10^−6^ m^2^ and Sa = πR^2^/2 = 1.57 × 10^−8^ m^2^ can be easily calculated. However, the antenna scale is much smaller than the wavelength (~ 1 mm). So *S*_*λ*_ = *πλ*^2^/4=7.8 × 10^−7^ *m*^2^ is taken for the active area. Hence the responsivity spectra at 1000 μW with polarization parallel (orange) and orthogonal (red) to the antenna are plotted in Fig. 8b. Obviously parallel measurement yielded much stronger intensity. which is typically due to much higher THz wave absorption rate of the antenna in parallel geometry. Notably, the peak responsivity of the parallel geometry curve reaches 0.1 V/W.

**Figure 9:**
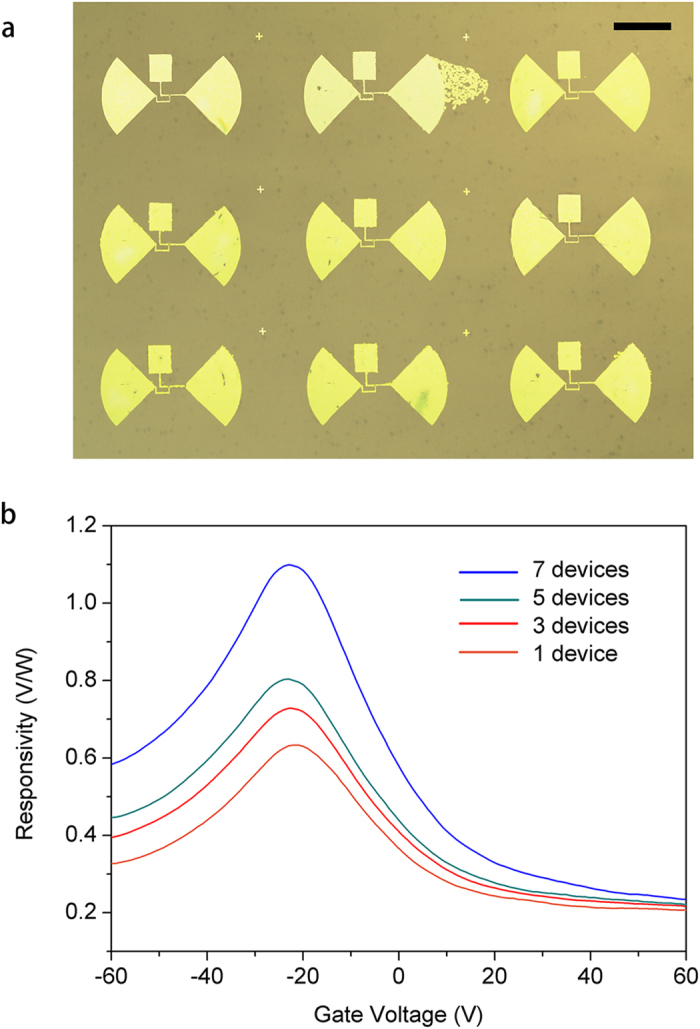
(**a**) Microscope image of 3 × 3 THz detectors array. Scale bar: 100 μm. (**b**) Responsivity curves of different number of individual detectors connected in series.

The current devices geometry’s tremendous advantage lays in the ability of realizing dense array of nanowires THz detectors. Hence the fabrication of pixel-like THz detectors array was done by larger-scale photolithography, transfer-printing and FIB electrodes deposition. A chip carrying 3 × 3 detectors is shown in the microscope image of Fig. 9a. Individual detector was designed the same way in Fig. 6. Wire bonding was performed on this chip and successfully enabled 7 operational devices. The active area of the chip is ~ 1.5 mm in size, which is on the same order of the incidence THz wavelength. On this chip, if more than two detectors are connected in series, it is naturally possible to render stronger photovoltage output. Responsivity characterization was performed, under the assumption that all incidence wave power were absorbed. The result for different number of devices connection is shown in Fig. 9b. Clearly, 7 devices together give the highest responsivity of 1.1 V/W. Notably, the change of responsivity intensity from 1 ~ 5 devices is not as large as from 5 ~ 7 devices, which is probably due to diffraction associated non-linear absorption phenomena. Nevertheless, the integration of multiple device not only produced stronger photoresponse, it paved the way for practical applications including focal plane pixel THz camera, high output photoconductive THz emitters, etc.

In summary, we have synthesized the InN nanowires and nano-necklaces on the Si substrate through the CVD process. The InN nanowires part was successfully aligned into micrometer sized trenches for controllable nanowire device fabrication. Then remarkable FET characteristics of aligned InN nanowires devices were presented and 99 cm^2^/VS electron mobility were achieved. The FET detector rendered 0.1 V/W peak responsivity, while multiple arrays of these detectors were able to be connected to increase the responsivity to 1.1 V/W. The results demonstrate that aligned InN nanostructures are promising for practical THz applications.

## Methods

### InN nanostructures growth

The InN nanostructures were synthesized in a horizontal three-temperature zones tube furnace system. A quartz boat containing 0.4 g of In powder (99.999%, Xiya Reagent) was placed in the middle of the II-temperature zone in the closed furnace. An 1.5 cm × 1.5 cm silicon substrate coated with a 60 nm gold colloid (BBI Solution) catalyst film was inversely placed on top of the quartz boat. In another word, the substrate with gold colloid side faced downward, in order to facilitate the growth of nanomaterials. The carrier gas, argon (99.999% purity, Messer), was introduced at one end of the quartz tube at a constant flow rate of 100 sccm (standard cubic centimeters per minute). The Ι and II temperature zones of the furnace were simultaneously increased to 700 °C in 30 min. Then the NH_3_ (99.99% purity, Messer) was introduced at same end of the quartz tube at a constant flow rate of 250 sccm when the setting temperature was reached. The growth was kept for 80 min. After the reaction the furnace was cooled to room temperature, and gray and black-colored products were found covering the silicon substrate.

### Materials characterization

The morphologies and microstructures of the as-synthesized products were systematically characterized. The XRD data was obtained with a Philips X’Pert PRO diffractometer equipped with Cu Ka radiation (λ = l.5418 Å). The accelerating voltage was set at 40 kV, with 40 mA flux in the 2Θ range of 10–90°. The field emission SEM images were obtained on the Model Uitra 55 from Carl Zeiss SMT Pte Ltd Co. Germany, which equipped with energy dispersive X-ray spectra (EDS). The TEM images were obtained on the Model Libra 200 PE (200kV) from Carl Zeiss SMT Pte Ltd Co. Germany.

### PL characterization

The room temperature spatial resolved PL spectra were carried out on a Model inVia Raman Microscope from RENISHAW Co. UK using a 514 nm laser as the excitation source.

### Nanowire alignment

First, device substrates (500 nm SiO_2_ on Si) were patterned by photolithography to define the trenches where nanowires will then be transferred. The trench on photoresist width and spacing can be adjusted by designing different photolithography plate; Second, a nanowire growth substrate was brought into contact with the patterned device substrate (nanowires facing the device substrate). A pressure of ~ 2 kg/cm^2^ was applied while the growth substrate was slide by several mm. After this, many of the nanowires will detach from growth substrate and transfer to device substrate by inter-molecular forces. Finally, the photoresist layer on device substrate was removed by acetone.

### THz detection characterization

The source used is a AV1450C (Vendor: CETC) signal generator (30 GHz) with frequency multipliers. The output wave is 290 GHz. The THz source is with adjustable power <1 mW and beam diameter of ~ 1.5 mm. The FET drain contact was connected to a current amplifier, which converting the current into a voltage signal. The output voltage was read by a lock-in-amplifier. The gate voltage was varied using a home built voltage meter. The detector was mounted on a motorized X–Y translation stage to maximize the measured photoresponse.

## Additional Information

**How to cite this article**: Chen, X. *et al.* Terahertz detectors arrays based on orderly aligned InN nanowires. *Sci. Rep.* **5**, 13199; doi: 10.1038/srep13199 (2015).
